# Early detection of cerebral microbleeds following traumatic brain injury using MRI in the hyper-acute phase

**DOI:** 10.1016/j.neulet.2017.06.046

**Published:** 2017-08-10

**Authors:** Tim P. Lawrence, Pieter M. Pretorius, Martyn Ezra, Tom Cadoux-Hudson, Natalie L. Voets

**Affiliations:** a Oxford Centre for Functional Magnetic Resonance Imaging of the Brain (FMRIB), Nuffield Department of Clinical Neurosciences (NDCN), University of Oxford, United Kingdom; b Department of Neurosurgery, Oxford University Hospitals NHS Foundation Trust, United Kingdom; c Department of Neuroradiology, Oxford University Hospitals NHS Foundation Trust, United Kingdom

**Keywords:** Traumatic brain injury, Traumatic cerebral microbleeds, Susceptibility weighted imaging, Hyper-acute

## Abstract

**Background:**

Traumatic brain injury (TBI) is a leading cause of death and disability in people under 45. Advanced imaging techniques to identify injury and classify severity in the first few hours and days following trauma could improve patient stratification and aid clinical decision making. Traumatic cerebral microbleeds (TCMBs), detectable on magnetic resonance susceptibility weighted imaging (SWI), can be used as markers of long-term clinical outcome. However, the relationship between TCMBs and injury severity in the first few hours after injury, and their natural evolution, is unknown.

**Methods:**

We obtained SWI scans in 10 healthy controls, and 13 patients scanned 3–24 h following TBI and again at 7–15 days. TCMBs were identified and total volume quantified for every lesion in each scan.

**Results:**

TCMBs were present in 6 patients, all with more severe injury classified by GCS. No lesions were identified in patients with an initial GCS of 15. Improvement in GCS in the first 15 days following injury was significantly associated with a reduction in microbleed volume over the same time-period.

**Conclusion:**

MRI is feasible in severely injured patients in the first 24 h after trauma. Detection of TCMBs using SWI provides an objective early marker of injury severity following trauma. TCMBs revealed in this time frame, offer the potential to help determine the degree of injury, improving stratification, in order to identify patients who require admission to hospital, transfer to a specialist center, or an extended period of intubation on intensive care.

## Highlights

• Traumatic cerebral microbleeds (TCMBS) can be identified using susceptibility weighted imaging in the first few hours after injury.
• TCMBs are a useful indicator of severity in this time frame.
• The presence of TCMBs is an early indicator of injury severity following trauma.
• There is a relationship between decreasing size of TCMBs and recovery.

## 1 Introduction

Traumatic brain injury (TBI) is the leading cause of death and disability in people under the age of 45 in developed countries. Long term clinical outcomes following injury are highly variable with significant physical and cognitive deficits experienced by patients with mild as well as severe TBI [1]. Accurately classifying severity of injury and predicting long term outcome in the first few hours after trauma (the hyper-acute phase) is often difficult, especially in mild and moderate injuries, as extent of injury may not be fully detected by computed tomography (CT). There is evidence that patients with moderate or severe TBI benefit from care in specialist neuroscience centers, and interventions to prevent hypoxia and hypotension [2], [3], [4], [5]. However, identifying which patients should be transferred to such centers and which patients require continuation of intensive care management is often difficult using current assessment tools available in the first few hours following injury. Consequently, there is an active search for more sensitive markers of injury that better stratify patients and identify varying pathophysiological patterns.

Traumatic cerebral microbleeds (TCMBs) identified using magnetic resonance susceptibility weighted imaging (SWI) have been reported to correlate with long-term clinical outcome [6]. While the number, location and size of the TCMBs have been linked to severity of injury and clinical outcome [7], [8], [9], [10], [11], [12], [13], as measured by the initial Glasgow Coma Scale (GCS) [14], other studies report no correlation between number of SWI lesions and outcome [15], [16]. As the optimal timeframe for image acquisition has not been extensively investigated, contrasting findings linking TCMBs with clinical outcome may have arisen partly due to variability in scan timing following injury, which ranged from days to years [17], [12]. In addition, the relationship between injury severity and TCMBs in the first day following trauma is unknown. The use of SWI to facilitate patient management is yet to be established.

Two recent preliminary studies have highlighted the potential for detection of TCMBs in the first few days after injury [18] [19]. The modest number of patients recruited provided only preliminary indication of a link between TCMB severity and clinical outcome. Both studies suggested that lesions could be identified, and will evolve, in the first week following trauma. One study suggested an increase in lesion volume and an apparent reduction in number due to lesion coalescence [18]; the other showed a reduction in lesion volume in the same timeframe [19]. The authors of the first study suggested that an increase in lesion volume could be explained by progressive microvascular damage. A different mechanism was postulated by the authors of the second study, who suggested that lesions decreased in size due to susceptibility differences relating to blood breakdown products, as proposed by Bradley and colleagues [20].

Here we performed a longitudinal MRI study in TBI patients to evaluate the incidence of TCMBs and their detectability in the first few hours following brain injuries of varying severity. We set out to test 1) the relationship between severity of injury (measured by initial GCS) and the presence, number and volume of TCMBs and 2) the relationship between change in GCS and evolution of TCMBs in the first 15 days following injury. We hypothesized that SWI scans acquired in patients with more severe injury (GCS 14 or less) would identify a greater number of TCMBs than scans in patients with better clinical status at presentation to hospital, and furthermore that improvements in patients’ clinical condition in the first few weeks of monitoring would be mirrored by reductions in total microbleed lesion volume.

## 2 Materials and methods

### 2.1 Participants

Adults over the age of 18 with TBI (mean age; 45 years, range; 23–83, 8 men, 5 women) were recruited prospectively from the Emergency Department of the Oxford University Hospitals NHS Foundation Trust. All patients received a CT scan as part of the standard trauma protocol (according to the NICE head injury guidelines [21]). Once life-threatening injuries were ruled out, patients were recruited for the study. Exclusion criteria included contraindications to MRI, other injuries leading to instability that would make scanning unsafe, or need for urgent surgery. MRI scans were performed within 24 h and again at 7–15 days following injury. In one patient, surgical intervention (fixation) for an upper limb fracture precluded repeat imaging at 7 days on safety grounds. The data from this participant were, therefore, only available from the first time-point. Clinical assessments including GCS and neurological examination were performed throughout the study time-period. None of the patients or controls (10 healthy controls, mean age; 30, range; 24–45, 4 men, 6 women, with no neurological or psychiatric history) received anti-coagulants, blood transfusions or clotting factors during the study period. The study was approved by the South Central − Berkshire Research Ethics Committee. Healthy controls provided informed written consent. All patients with capacity at the time of initial recruitment gave written informed consent. For patients lacking capacity, the lead clinician, in consultation with the family, signed a declaration form to confirm agreement for the patient to be recruited into the study. Explicit patient consent was sought as soon as possible upon recovery.

### 2.2 MRI data acquisition

All imaging data were acquired on a 3T Siemens Magnetom Verio scanner at the Oxford Acute Vascular Imaging Centre (AVIC). The scanning protocol included T1-weighted MPRAGE, T2-weighted Turbo spin echo and T2* SWI structural imaging sequences. SWI sequences were acquired with the following parameters: TR = 27 ms, TE = 20 ms, 64 slices providing whole-brain coverage, GRAPPA acceleration factor = 2, voxel dimensions = 0.9 × 0.9 × 2 mm, duration = 3:22 min.

### 2.3 Quantification of traumatic cerebral microbleeds

TCMBs were identified on each scan by a fellowship trained consultant neuroradiologist, blinded to clinical history (i.e. whether each scan was from a patient or control). For each scan, the neuroradiologist noted the location and size of all hypointense lesions in each SWI scan. A lesion was considered to be a TCMB if it had the following characteristics on SWI: predominantly hypointense, within parenchyma, curvilinear or oval, separate from artefact, and when close to or in contact with a vein blunt endings seen to distinguish it from a vessel. T1 and T2 images were used to help identify lesion location, although the lesions were not directly visible in these sequences. Subsequently, each lesion was manually delineated in Fslview (part of the FMRIB software library, www.fmrib.ox.ac.uk/fsl, Oxford, UK) [22] creating a binary mask. The number of voxels included in the mask was multiplied by the voxel volume (0.9 × 0.9 × 2.0 mm) to give the overall lesion volume. The total number of voxels and the total voxel volume were calculated for each lesion allowing for total lesion volume per scan to be quantified.

### 2.4 Statistical analysis

Statistical analyses were performed using SPSS (v24). GCS scores and total recorded TCMBs were not normally distributed. Therefore tests for correlations between injury severity (GCS) and total TCMB lesion volume were performed using Spearman’s two-tailed correlations. A Wilcoxon signed rank test was used to determine differences in total TCMB volume between the first and second scans.

## 3 Results

In our prospective cohort, traumatic cerebral microbleeds were identified in 6/13 patients and 0/10 healthy controls (Table 1). The greatest lesion volume was seen in patients with an initial GCS <9. All patients with TCMBs detected during the hyper-acute phase had a presenting GCS of 14 or less. Lesions were located, in order of decreasing frequency, in the frontal, temporal, parietal and occipital lobes, predominantly at the grey-white matter interface of the frontal lobes. Lesions were also identified in the splenium of the corpus callosum, in the putamen and globus pallidus, thalamus, cerebral peduncles and midbrain tegmentum.

**Table 1:** Imaging and clinical assessment of study patients at Day 0 and between days 7–15. Table 1 Legend. Table displaying the total traumatic cerebral microbleed (TCMB) volume and number per scan, additional injuries seen on imaging, scan timing and Glasgow Coma Scale (GCS) scores. Patient 8 was precluded from undergoing follow-up scanning at 1 week due to an intervening operation involving metallic implants to fix an upper limb fracture. IVH = intraventricular hemorrhage, SDH = subdural hematoma, EDH = extradural hematoma, TSAH = traumatic subarachnoid hemorrhage.

Patient	Scan 1 Total vol.	Scan 2 Total vol.	Scan 1 no. of lesions	Scan 2 no. of lesions	Other traumatic intracranial lesions (not included in lesion volume)	Scan 1 timing	Scan 2 timing	GCS at day 0	GCS at day 7–15
1	249	195	6	6		21 h	8 days	7	15
2*	3415	4247	17	17	IVH	3 h	7 days	6	6
3	74	47	2	2	SDH	12 h	7 days	14	15
4	60	55	2	2		18 h	7 days	14	15
5	0	0	0	0		3 h	15 days	15	15
6	1051	869	12	12		15 h	9 days	8	15
7	0	0	0	0		3.5 h	7 days	15	15
8	0	X	0	X		5 h	N/A	14	15
9	0	0	0	0	EDH	21 h	7 days	15	15
10	0	0	0	0		3.5 h	7 days	14	15
11	0	0	0	0		4 h	7 days	15	15
12	0	0	0	0		15 h	7 days	14	15
13	6884	3038	35	33	IVH, TSAH, SDH	6 h	7 days	3	15

* Patient 2 displayed variation to the trend of decrease in total lesion volume explained by expansion of one lesion, visible on CT, representing a small hematoma.

**Fig. 1:**
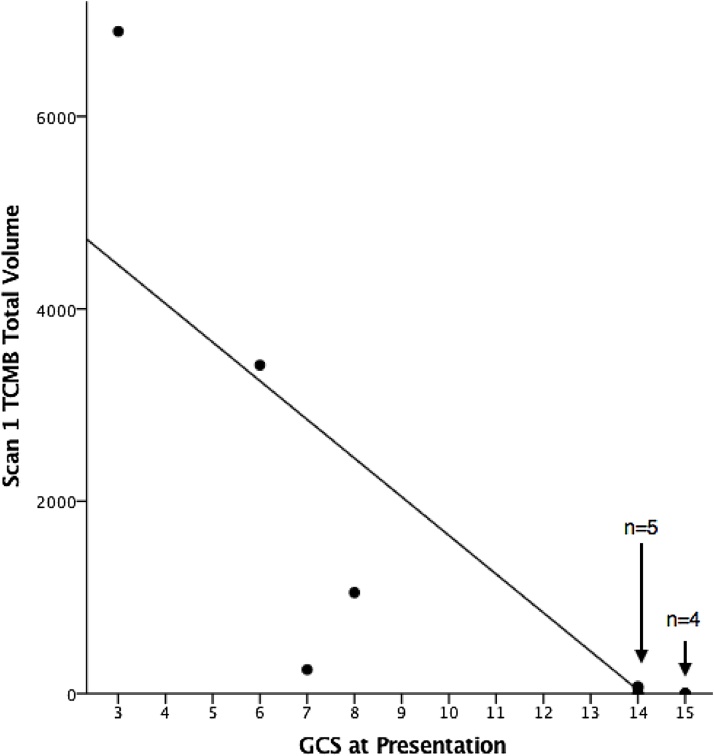
Relationship between initial Glasgow Coma Scale (GCS) and total lesion volume identified on the first scan. Figure 1 Legend. Graphical representation of the relationship between GCS at presentation and total TCMB lesion volume on the first scan. Patients with a poor GCS at presentation ( < 9) all had lesions identified with SWI. Two patients with a GCS of 14 at presentation had lesions identified with SWI. These data points are over-lapping. All other data points for patients presenting with a GCS of 14 or 15 are represented by over-lapping data points. No patients with a GCS of 15 at presentation had lesions identified. A negative correlation was seen between initial GCS and total lesion volume on the first scan (day 0) (R = −0.867, p < 0.001).

The total volume of lesions negatively correlated with severity as assessed by the initial GCS (Fig. 1). Greater TCMB volume on the first scan was associated with lower GCS score at presentation (R = −0.867, p < 0.001). No patients with a presenting GCS of 15 had any detectable SWI lesions on the first scan.

**Fig. 2:**
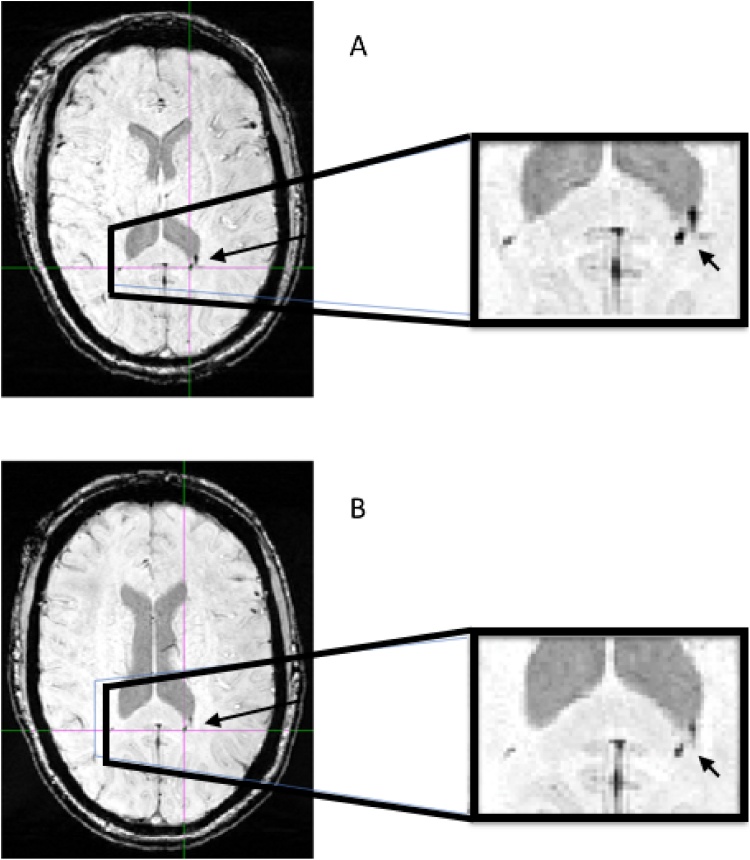
Susceptibility-weighted MR images illustrating change in volume in patient 13. Figure 2 Legend. Representative axial images with magnified views of the splenium of the corpus callosum from patient 13 in whom a decrease in total traumatic cerebral microbleed (TCMB) volume was seen between the first (A) and second (B) scan, 7 days apart. This was accompanied by an increase in GCS and marked improvement in clinical state between the two time-points.

**Fig. 3:**
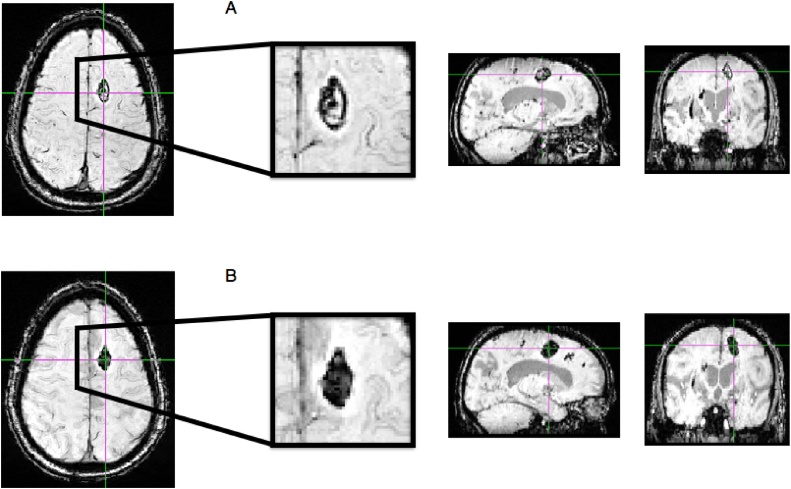
Susceptibility-weighted MR images illustrating change in volume in patient 2. Figure 3 Legend. Representative axial, coronal and sagittal images with magnified axial views of the left superior frontal gyrus region from patient 2 in whom an increase in total traumatic cerebral microbleed (TCMB) volume was seen between scan 1 (A) (1819 mm^3^) and scan 2 (B) (2845 mm^3^), 7 days apart. This patient did not experience an improvement in GCS between scans 1 and 2 and their overall clinical state worsened.

Within the group of 6 patients who had TCMBs on their presenting SWI scan, the lesions appeared to decrease in volume between the first ( < 24 h) and second scan (7–15 days) with the exception of one patient. In our sample the total volume change between these time-points did not reach significance (p = 0.249). In 5/6 patients, there was a reduction in the size of lesions identified between the first and second scans. In one case there was a reduction in number of lesions. Reduction in volume between scan 1 and 2 in a representative patient is shown in Fig. 2. Analysis of the 5 patients who displayed a reduction in volume, excluding patient 2, did reach significance (p = 0.043). Only one patient (patient 2) showed an increase in overall lesion volume, due to expansion of one large lesion (representative patient illustrating apparent increase in volume between scan 1 and 2 shown in Fig. 3). This single lesion, which was visible on CT, probably represents a small hematoma (1819 mm^3^ at scan 1 (3 h after injury), 2845 mm^3^ at scan 2 (7 days after injury)). No new lesions were identified between the first and second scan in any patient. In one patient there was a reduction in number of lesions from 35 on the first scan to 33 on the second.

**Fig. 4:**
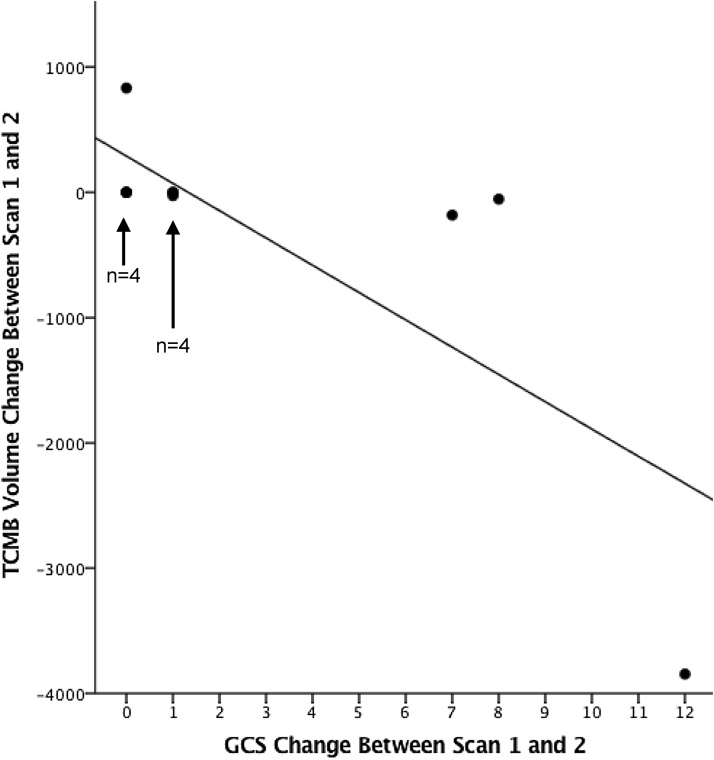
Relationship between change in GCS from presentation to second scan and change in total lesion volume between scan 1 and scan 2. Figure 4 Legend. Graphical representation of the relationship between change in GCS from presentation to the second scan and change in total lesion volume between scan 1 and scan 2. Patients who experienced the most marked improvement in GCS had the greatest reduction in total lesion volume between the two scans. There was a negative correlation between GCS change and total SWI lesion volume change (R = −0.718, p = 0.009). Data points for patients with no change, or very little change, in lesion volume are represented by over-lapping data points due to an equivalent change in lesion volume value.

An improvement in the GCS (in the first 7–15 days) was correlated with a fall in total lesion volume, with the greatest reduction seen in patients displaying the most marked improvement in GCS (R = −0.860, p < 0.001) (Fig. 4). A contrasting pattern is represented in patient 2 whose total lesion volume increased, primarily due to the expansion of one relatively large lesion (visible on CT). This patient remained intubated and ventilated with no change in GCS and a worsening clinical picture.

## 4 Discussion

Previous studies, predominantly in the pediatric population, have highlighted the potential of susceptibility weighted imaging (SWI) to assess the severity of injury and long term clinical outcome following traumatic brain injury (TBI) [6], [7], [8], [9]. These studies do not reveal the optimum time following injury to carry out imaging to acquire SWI data or whether lesions can be identified in the time-period when clinical decisions are being made regarding patient care (the hyper acute phase). Two initial small-sampled studies indicate that there may be variability in SWI findings depending on the point at which data are acquired [18], [19].

Here, we demonstrated a relationship between the severity of injury (as measured by GCS) and the presence, and volume, of TCMBs identified in the first day following trauma. Those patients who had a good GCS (of 15) at the outset, and remained well following their injury, had no lesions, whereas patients with more severe injuries were found to have lesions on their initial SWI scans undertaken within 24 h of injury. Importantly, some patients with a GCS of 14 were found to have TCMBs on their presenting MRI scan. This finding offers potential clinical value in that, early identification of the presence, and volume, of lesions provides information regarding *severity* that could aid clinical decision making, and that the absence of lesions could be an important *discriminator* when considering discharge from the emergency department (ED) rather than admission to hospital. It has already been shown in previous studies that lesion number and volume is linked to severity and outcome [7], [8], [9], [10], [11], and our findings confirm this, but the existence of TCMBs at such an early time point has not previously been shown beyond small case series. We show that a correlation can be seen between initial injury severity and the presence of TCMBs when imaging is performed as soon as possible after injury. This raises the potential to use SWI as an adjunct to GCS and CT but also as a vital tool when CT is normal and GCS is not assessable.

The observation of early MRI evidence of microbleeds (within hours of injury) correlated with injury severity has a potentially significant bearing on where and how patients are managed in the first few days after trauma, when GCS and CT alone are often insufficient to identify patients who may benefit from an escalation of care. The advancement of prehospital care has led to an improvement in prevention of secondary brain injury, utilizing early intubation and rapid fluid resuscitation with blood to avoid hypoxia and hypertension, respectively. Consequently, an increasing number of patients now arrive in the ED intubated following a TBI. Clinicians need to determine the degree of brain injury, often with no evidence of TBI on CT and an incomplete GCS examination, in order to make decisions regarding the next stage of care. The demonstrated sensitivity of SWI to detect TCMBs could help identify patients who would benefit from further sedation and continued intensive care management, and those whose brain injury is mild enough to warrant early extubation. The appropriate timing of SWI data acquisition is therefore crucial, as these decisions need to be made early.

In our data, the overall number of lesions was stable between the two time-points, with only one patient showing evidence of a fall in number. Furthermore, the change in volume over time in all 6 patients was not significant. In one patient, lesion volume expansion was driven by one larger lesion possibly representing a small hematoma. In the remaining 5/6 patients, TCMB total volume fell over the time-period studied. As it is possible TCMB volume may change between the two time-points, the timing of MRI scans should be taken into account when using SWI as a modality to assess injury severity. Our findings support the use of early scans (within 24 h), however, further studies are needed, investigating multiple time-points within the first few days, to identify the optimal window.

Although the reduction in lesion volume between the two time-points was not significant in our population, greater improvement in GCS was associated with greater reduction in lesion volume between 24 h and 1–2 weeks after injury. This finding implies that it is not merely the presence of post-traumatic microbleeds in the first few hours after injury, but also their evolution over subsequent days, that may be important for assessing severity and potentially predicting clinical outcome. It is evident that TCMBs identified using SWI after head trauma evolve, but the direction of this evolution has been inconsistent across studies. In our patient group, the majority of TCMBs decreased in volume in the days after trauma, although this finding was only significant when patient 2 (expanding hematoma) was removed from the analysis. Toth et al. [18] reported an increase in lesion volume following trauma explained by possible microvascular failure, whereas, Watanabe et al. [19] identified a decrease in volume that they proposed was due to evolution of the susceptibility signal related to degradation of blood. One possible explanation for such variability is that evolution of TCMBs may be linked to initial severity and subsequent progression of injury. Indeed, although our data show a reduction in lesion volume, there is evidence from patient 2 that some lesions expand. However expansion in lesion volume may have another explanation. The changes in lesion volume identified with SWI could be an artefact of the imaging modality. A blooming effect may occur depending on the state of the blood degradation. This effect is most pronounced with hemosiderin, present in the chronic phase following injury. Therefore the lesion expansion (in patient 2) could reflect the changes in physical properties of the blood products associated with the different stages of breakdown, rather than a true expansion of the hematoma.

The SWI lesions clearly provide some marker of brain injury, without however giving us a detailed window onto the mechanism by which this injury arose. While this marker strongly correlated with initial GCS and severity, we do not intend to imply causation. Only an associative relationship can be interpreted from our results. Combining SWI with other imaging modalities such as magnetic resonance spectroscopic imaging (MRSI), diffusion tensor imaging (DTI) and resting state functional MRI (rsFMRI) may provide additional information regarding mechanism.

## 5 Limitations

There are a number of limitations that need to be addressed in this paper. Firstly the small number of patients and degree of injury heterogeneity could cause the results to be driven by possible outliers. In order to understand whether the results were influenced by the extreme change in GCS of patient 12, the analysis of GCS change associated with TCMB volume change was repeated with exclusion of this patient. The findings remained significant (R = −0.619 and p = 0.042). The overall study numbers remain small, and the range of injury severity large, therefore a more extensive study is needed to confirm our findings.

Secondly the calculation of TCMB volume is susceptible to measurement errors, especially for small lesions. This may be an important factor in assessing the change in lesion volume between two time points. Automated systems for TCMB identification and analysis are being developed and provide potential for more accurate assessment of lesions [23], [24].

Thirdly, the age range in the patient group was wider than in the control group. Cerebral microbleeds are more commonly found in the older population (35.7% in the > 80 group) [25]. The absence of microbleeds seen in our control population could be due to the relatively young age of the control subjects.

Small sample size, injury heterogeneity and possible small measurement errors do limit the generalizability of our findings.

In conclusion, our study has shown that TCMBs can be identified in severely injured TBI patients using MR SWI within the first few hours after trauma. Our results demonstrate a relationship between injury severity, measured by GCS, and total lesion volume, measured by SWI, in the first day following injury, with no lesions seen in the milder patients and multiple lesions seen in the more severely injured patients. Long-term outcome results from our cohort will reveal if there is a relationship between TCMBs identified within the first day and clinical outcome at 6 months. These data acquired in 13 patients with varying degrees of TBI adds to the understanding of how TCMBs behave in the first few days following injury. Direct associations with clinical performance and outcome in the first weeks suggest that the presence of TCMBs detected on SWI could improve the classification of patients beyond that provided by CT and GCS. In particular, hyper-acute MRI may prove useful to guide clinical decisions in the first day after injury, for example by providing an indicator of patients who would benefit from admission to hospital, escalation of care and continuation of intensive care management. Future studies using structural imaging to assess white matter tracts may expand our understanding of the link between TCMBs and diffuse axonal injury (DAI), while measures of the brain’s functional connectivity could inform possible links between SWI lesion location and cognitive deficits potentially providing the opportunity to use SWI, in the hyper-acute phase, as a tool for predicting not only outcome but specific deficits.

## Funding

Supported by the NIHR Biomedical Research Centre, based at Oxford University Hospitals Trust, Oxford. The views expressed are those of the author(s) and not necessarily those of the NHS, the NIHR or the Department of Health. This work was supported by core funding from the Wellcome Trust [203139/Z/16/Z].
